# The association between cadmium and lead exposure and blood pressure among workers of a smelting industry: a cross-sectional study

**DOI:** 10.1186/s40557-017-0202-z

**Published:** 2017-10-04

**Authors:** Hyun Chan An, Joo Hyun Sung, Jiho Lee, Chang Sun Sim, Sang Hoon Kim, Yangho Kim

**Affiliations:** 1 Department of Occupational and Environmental Medicine, Ulsan University Hospital, University of Ulsan College of Medicine, 877, Bangeojinsunhwando-ro, Dong-gu, Ulsan, 44033 Republic of Korea; 2 Department of Occupational and Environmental Medicine, Gyeongsang National University Changwon Hospital, Gyeongsang National University School of Medicine, Ulsan, Republic of Korea

**Keywords:** Cadmium, Lead, Blood pressure

## Abstract

**Background:**

Lead and cadmium are harmful heavy metals that are used for a variety of occupational purposes. Considering their potentially hazardous effects on health, studies on the association between exposure to these two heavy metals and health disorders have been actively conducted. This study aimed to determine the association between blood lead and cadmium levels and blood pressure in workers exposed to lead and cadmium in a smelter.

**Methods:**

Nine hundred and sixty-three male workers who worked in a smelter, and underwent medical examinations at the Ulsan University Hospital between January 1 and December 31, 2014, were selected as participants. Among them, 310 subjects whose data on height, weight, blood pressure, fasting blood glucose, lipid profile, and blood lead and cadmium levels were available and who answered the questionnaire were selected as the final participants. We investigated the drinking habit, smoking status, exercise adequacy, and family history of hypertension of these workers using formal questionnaires. A statistical analysis was conducted using Student’s *t*-test, analysis of variance, and linear or logistic regression.

**Results:**

The association between blood lead and cadmium levels and blood pressure was analyzed through statistical adjustment of the risk factors of hypertension. Results showed an association between blood cadmium level and blood pressure elevation. However, blood lead level was found to be not correlated with blood pressure elevation.

**Conclusions:**

This study shows the association between blood cadmium level and systolic blood pressure (SBP) and diastolic blood pressure (DBP) elevation.

**Trial registration:**

IRB No. 2017–03-037. Retrospectively Registered 30 March 2017.

## Background

Although lead and cadmium are prevalent heavy metals in the environment, exposure to these metals primarily occurs as a result of human activities [1], particularly for lead and cadmium, which are widely used in various industries [2]. For example, lead is used in mining, smelting and refining, printing, and battery manufacturing, and cadmium is utilized in smelting and refining, plastic and battery manufacturing, and alloy production [3–6].

These two heavy metals mainly enter the body through the respiratory and digestive systems [7–9], and they accumulate and remain for a long time. The half-lives of lead and cadmium are 5–20 years in the bones and 10–30 years in the kidneys, respectively [10–12]. Given the health hazards caused by these heavy metals, studies on lead or cadmium exposure and health impairment are continuing. There are studies on renal [13, 14], hematopoietic [2, 15, 16], and cardiovascular [17, 18] systems effects due to lead exposure, and there are studies on renal [19, 20], cardiovascular [21], and musculoskeletal [22] systems effects due to cadmium exposure.

In particular, numerous studies have been conducted on the relationship between lead or cadmium exposure and blood pressure elevation. Although some studies reported the relationship between lead exposure and blood pressure elevation [20, 23–25], other researches revealed the absence of association between lead exposure and blood pressure elevation [26–28]. The effects of lead exposure on blood pressure are inconsistently reported, and varied depending on the age and gender of the participants within the same study [29]. The same inconsistent findings were also reported on the association between cadmium exposure and blood pressure elevation. Some studies revealed the associations between cadmium exposure and elevated blood pressure [30–32], but other research also reported opposite results [33–35].

Hypertension is a highly prevalent disease that causes complications in major organs, such as the brain, heart, and kidneys [36]. The risk factors for blood pressure elevation generally include increasing age, high body mass index, frequent drinking, lack of physical activity, smoking, stress, diabetes, dyslipidemia, race [37–43], and numerous researches related to these risk factors have been conducted. However, only few studies have been performed on the association between blood pressure and exposure to heavy metals in Korea [24, 25]. Furthermore, study subjects of the previous papers were general population who had lower exposure to the metals, but not workers who were exposed to heavy metals occupationally. Therefore, in this study, we aimed to confirm the association between lead and cadmium exposures and blood pressure increase in workers simultaneously exposed to these two heavy metals.

## Methods

### Study subjects

Nine hundred and sixty-three male workers who worked in a copper smelter, and underwent medical examinations at the Ulsan University Hospital between January 1 and December 31, 2014, were selected as participants. The copper smelter produces purified copper from copper ore through various processes such as drying, smelting, and refining. All subjects in our study were production workers who worked in mechanical maintenance, refining, smelting furnace processes, and others. They were exposed to lead and cadmium as well as copper generated in the process. We analyzed the participants’ age, body mass index, work period, drinking habit, smoking status, exercise adequacy, family history of hypertension, blood lead and blood cadmium levels, and other related factors based on the results of the medical examinations and their answers to the questionnaires. Among all the participants, 310 workers whose data were complete and who were exposed to lead and cadmium as hazardous factors were selected as the final analysis subjects.

### Basic data collection

The ages and work periods of the participants were calculated based on their date of birth and start date of occupation, respectively. High-risk alcohol drinking habit was defined as alcohol consumption of more than 8 glasses per day [44], and the participants were classified based on their smoking status as current nonsmokers (i.e., nonsmokers and ex-smokers) and current smokers [45]. Adequate exercise was defined as performance of moderate physical activities, such as playing tennis doubles or very brisk walking for more than 30 min a day and three or more times a week. A positive family history of hypertension was defined as having parents or siblings who were diagnosed with hypertension or taking an antihypertensive drug [46]. The weight and height were measured using an auto weight and height measuring system (GL-150; G-Tech International Co., Ltd., Korea), and body mass index was calculated by dividing the body weight (kg) by the square of the height (m^2^). Blood pressure was measured once in the brachial artery of subjects, while subjects were seated following 5-min rest period. Hypertension was defined as DBP of at least 90 mmHg or SBP of at least 140 mmHg or self-reported current use of an anti-hypertensive medication. The SBP and DBP were measured using an automatic blood pressure monitoring equipment (FT-500R; Jawon Medical Co., Ltd., Korea). Dyslipidemia defined by either a fasting total cholesterol level of ≥200 mg/dL, or low density lipoprotein cholesterol ≥130 mg/dl, or high density lipoprotein cholesterol ≤ 40 mg/dL [47]. Blood glucose and lipid profile were measured after 12-h fasting.

### Determination of lead and cadmium in whole blood

The blood lead and cadmium levels were pretreated using a dilution method and analyzed with a graphite furnace atomic absorption spectrometer (240Z; Agilent Technologies, Santa Clara, CA, USA). The blood lead level was analyzed by mixing 50 μl whole blood, 50 μl distilled water, and 900 μl diluted solution (1% Triton X-100/0.2% (NH_4_)_2_HPO_4_) in a tube. The blood cadmium level was analyzed by mixing 150 μl whole blood, 150 μl distilled water, and 1700 μl diluted solution (1% Triton X-100/0.2% (NH_4_)_2_HPO_4_) in a tube. Blood lead and cadmium concentrations were measured at Ulsan University Hospital, which passed the Quality Assurance Program operated by the Korean Occupational Safety and Health Agency, and is certified by the Korean Ministry of Employment and Labor as a designated laboratory for analysis of specific chemicals, including heavy metal and certain organic chemicals.

### Statistical analysis

The data collected from the 310 subjects were statistically analyzed using SPSS 21.0 (IBM SPSS Inc., Chicago, IL, USA). The blood lead and cadmium levels were analyzed through logarithmic (base 2) transformation because they were not normally distributed and were right skewed. The heavy metal levels in the blood were expressed as the geometric mean (GM) and 95% confidence interval (CI). Student’s *t*-test and analysis of variance were used for the comparison of the mean values. Simple linear regression analysis was utilized to confirm the association between SBP and DBP and blood heavy metal levels. In linear regression, 292 subjects were analyzed excluding those who were taking antihypertensive drugs, because the medications might affect SBP and DBP. Multiple linear regression analysis was performed to adjust the relevant factors that could affect the blood pressure and determine the relationship between blood lead and cadmium concentrations and blood pressure elevation. Risk factors for hypertension such as age, body mass index, dyslipidemia, diabetes, alcohol drinking habit, smoking status, exercise habit, and family history of hypertension were included as covariates. Stress was not available in this study. We also evaluated the relationship of blood lead and cadmium concentrations with blood pressure elevation by stratifying study subjects into those younger than 40 years and those aged 40 years or over. In addition, odds ratios (ORs) and 95% CIs for hypertension were calculated for log_2_-transformed blood lead and cadmium after adjustment of the same covariates as in the logistic regression analyses.

Significance level was set at *p* < 0.05; a *p*-value less than 0.05 indicated statistical significance.

## Results

Three hundred ten subjects were included in the final analysis. The variables that could affect the blood pressure and blood lead and cadmium levels were presented using GM and 95% CI. The age range of the subjects was from 21 to 61 years. The workers were divided based on their ages, that is, 20–29 years, 30–39 years, 40–49 years, and 50 or older, and the blood lead and cadmium levels of these age groups were then determined. The GMs of the blood cadmium levels of the total and the four age groups were 1.053, 0.655, 1.036, 1.172, and 1.347 μg/L, respectively. The blood cadmium concentration significantly increased with age (*p* < 0.001). However, no statistically significant relationship was found between blood lead levels and age.

The subjects were also divided into groups based on their work periods, that is, less than 10 years, 10–19 years, and more than 20 years, and their blood lead and cadmium levels were then analyzed. The GMs of the blood cadmium concentrations of the three groups were 0.829, 1.193, and 1.399 μg/L, respectively, and the blood cadmium levels significantly increased as the work period increased (*p* < 0.001). However, no statistically significant association was noted between blood lead levels and work period.

The GMs of blood cadmium levels were 0.965, 1.126 μg/L for group without dyslipidemia and dyslipidemia group, respectively, and the difference was statistically significant (*p* = 0.039). However, a statistically significant difference in blood lead levels was not found between the two groups.

The subjects were further divided based on their smoking status into current nonsmoker (i.e., nonsmoker and ex-smoker) and current smoker groups. The GMs of the blood lead levels were 5.232 and 6.385 μg/dL for the current nonsmoker and current smoker groups, respectively, and the difference was statistically significant (*p* < 0.001). Additionally, the GM of the blood cadmium levels of the current smoker group was 1.155 μg/L, which was higher than that of the current nonsmoker group (0.972 μg/L), and the difference was statistically significant.

Furthermore, body mass index, fasting blood glucose, high-risk alcohol drinking habit, adequate exercise, and family history of hypertension were found to be not significantly associated with blood lead and cadmium levels (Table 1).

**Table 1:** General characteristics of subjects

Classification variables		Number	Geometric mean (95% CI)	
			Blood lead (ug/dL)	Blood cadmium (ug/L)
Total		310	5.839 (5.421–6.068)	1.053 (0.980–1.131)
Age group (years)	20 ~ 29^(a)^	57	4.991 (4.313–5.774)	0.655 (0.529–0.812)^‡,c^
	30 ~ 39^(b)^	86	5.792 (5.217–6.431)	1.036 (0.933–1.150)
	40 ~ 49^(c)^	99	5.961 (5.379–6.605)	1.172 (1.055–1.303)
	≥ 50^(d)^	68	6.017 (5.385–6.722)	1.347 (1.187–1.575)
Work period (years)	< 10^(e)^	145	5.698 (5.243–6.192)	0.829 (0.739–0.930)^‡,d^
	10 – 19^(f)^	77	5.928 (5.230–6.718)	1.193 (1.071–1.328)
	≥ 20^(g)^	88	5.632 (5.109–6.209)	1.399 (1.258–1.555)
BMI (kg/m^2^)	< 23	90	5.747 (5.149–6.415)	1.036 (0.903–1.189)
	23–24.9	85	5.567 (5.006–6.190)	1.067 (0.927–1.228)
	≥ 25	135	5.836 (5.359–6.356)	1.055 (0.949–1.173)
Fasting blood glucose (mg/dL)	≤ 99	229	5.851 (5.489–6.236)	1.032 (0.948–1.123)
	100–125	68	5.357 (4.675–6.139)	1.140 (0.992–1.311)
	≥ 126	13	5.768 (4.471–7.441)	0.985 (0.633–1.534)
Dyslipidemia	No	135	5.424 (4.958–5.932)	0.965 (0.865–1.078)^‡^
	Yes	175	5.988 (5.571–6.436)	1.126 (1.026–1.235)
High risk alcohol drinking habit^a^	No	182	5.900 (5.465–6.370)	1.010 (0.889–1.149)
	Yes	128	5.509 (5.071–5.984)	1.083 (0.997–1.176)
Smoking status	Current non smoker	167	5.232 (4.856–5.637)^‡^	0.972 (0.875–1.080)^‡^
	Current smoker	143	6.385 (5.875–6.939)	1.155 (1.052–1.268)
Adequate exercise^b^	No	141	5.726 (5.292–6.197)	1.003 (0.894–1.125)
	Yes	169	5.743 (5.299–6.223)	1.096 (1.002–1.199)
Family history of hypertension	No	277	5.739 (5.403–6.096)	1.046 (0.970–1.128)
	Yes	33	5.705 (4.845–6.717)	1.114 (0.885–1.402)

*p* - value was calculated by Student *t*-test, analysis of variance. ^a^Drink more than 8 glasses per day. ^b^Exercise harder than moderate physical activity at least 30 min per day and 3 times per week. ^‡^ *p* < 0.05, ^c^post hoc comparison: (a) < (b), (c), (d), ^d^post hoc comparison: (e) < (f), (g)

The SBP and DBP of the final subjects were measured and examined using simple linear regression analysis to determine the association between blood pressure elevation and blood lead and cadmium levels. Blood lead and cadmium levels were analyzed using logarithm (base 2) transformation. SBP and DBP were significantly associated with log_2_ blood cadmium levels (*p* = 0.004 and *p* = 0.001, respectively). The results indicated that a doubling of blood cadmium was associated with the increase in SBP and DBP by 2.310 mmHg (95% CI: 0.727–3.893 mmHg) and 2.067 mmHg (95% CI: 0.814–3.319 mmHg), respectively. However, no statistical association was detected between SBP and DBP and blood lead levels (Table 2).

**Table 2:** Relationship between blood pressure and independent variable

Variables	β coefficient (Standardized β coefficient)	95% CI	R^2^	*p*-value
Systolic blood pressure				
Per doubling of blood cadmium^a^	2.310 (0.166)	0.727–3.893	0.028	*0.004* ^†^
Per doubling of blood lead^a^	0.344 (0.019)	- 1.715 – 2.403	0.000	0.742
Diastolic blood pressure				
Per doubling of blood cadmium^a^	2.067 (0.187)	0.814–3.319	0.035	*0.001* ^†^
Per doubling of blood lead^a^	- 0.300 (− 0.021)	- 1.936 – 1.335	0.000	0.718

*p* - value was calculated by simple linear regression analysis. ^a^logarithm (base 2) transformation of blood cadmium or lead concentration. Those who were taking antihypertensive drugs were excluded. ^†^ *p* < 0.05

The relationship between elevated blood pressure and blood lead and cadmium levels were examined with regard to age, body mass index, fasting blood glucose, dyslipidemia, drinking habit, smoking status, exercise adequacy, and family history of hypertension, which may affect the blood pressure of the participants, using multiple linear regression analysis. SBP was significantly associated with body mass index and per doubling of blood cadmium levels (*p* < 0.001 and *p* = 0.012, respectively). The results indicated that a doubling of blood cadmium was associated with the increase in SBP by 2.171 mmHg (95% CI: 0.487 - 3.854 mmHg). Other variables, such as age, fasting blood glucose, dyslipidemia, high-risk alcohol drinking habit, smoking status, adequate exercise, family history of hypertension, and log_2_ blood lead levels, were not statistically significant (Table 3).

**Table 3:** Relationship between systolic blood pressure and other independent variables

Independent variables	β coefficient (Standardized β coefficient)	95% CI	*p*-value
Age	0.061 (0.082)	- 0.101 – 0.222	0.461
BMI	1.509 (0.230)	1.056–1.961	*< 0.001* ^†^
Fasting blood glucose	0.044 (0.044)	- 0.042 – 0.131	0.315
Dyslipidemia (Yes vs. No)	- 1.014 (1.497)	- 3.961 – 1.934	0.499
High risk alcohol drinking habit (Yes vs. No)	- 0.851 (1.412)	- 3.630 – 1.929	0.547
Smoking status (Yes vs. No)	- 1.244 (1.446)	- 4.091 – 1.602	0.390
Adequate exercise (Yes vs. No)	- 2.324 (1.396)	- 5.073 – 0.425	0.097
Family history of hypertension (Yes vs. No)	4.066 (2.304)	- 0.469 – 8.601	0.079
Per doubling of blood cadmium^a^	2.171 (0.855)	0.487–3.854	*0.012* ^†^
Per doubling of blood lead^a^	- 0.636 (1.029)	- 2.661 – 1.389	0.537
R^2^ = 0.212 (*p* *< 0.001*)			

*p* - value was calculated by multiple linear regression analysis. ^a^logarithm (base 2) transformation of blood cadmium or lead concentration. Those who were taking antihypertensive drugs were excluded. ^†^ *p* < 0.05

DBP was significantly associated with age, body mass index, family history of hypertension, and per doubling of blood cadmium levels (*p* = 0.002, *p* < 0.001, *p* = 0.008, and *p* = 0.029, respectively). The results indicated that a doubling of blood cadmium was associated with the increase in DBP by 1.462 mmHg (95% CI: 0.147–2.777 mmHg). Other variables, such as fasting blood glucose, dyslipidemia, high-risk alcohol drinking habit, smoking status, adequate exercise, and log_2_ blood lead levels, were not statistically significant (Table 4). In addition, multiple linear regression analysis with age stratification showed that doubling of blood cadmium levels was significantly associated with the increase in SBP only by 3.336 mmHg (95% CI: 0.572–6.099 mmHg) in subjects aged 40 years or older after adjustment for the same covariates (*p* = 0.018). There was no significant association in subjects younger than 40 years (Table 5). We also analyzed the association of blood cadmium and blood lead levels with hypertension using logistic regression analysis. But there was no association of the presence of hypertension with blood cadmium and lead levels (data not shown).

**Table 4:** Relationship between diastolic blood pressure and other independent variables

Independent variables	β coefficient (Standardized β coefficient)	95% CI	*p*-value
Age	0.202 (0.064)	0.075–0.328	*0.002* ^†^
BMI	0.950 (0.180)	0.597–1.303	*< 0.001* ^†^
Fasting blood glucose	0.062 (0.034)	- 0.006 – 0.129	0.074
Dyslipidemia (Yes vs. No)	0.167 (1.169)	- 2.134 – 2.469	0.886
High risk alcohol drinking habit (Yes vs. No)	- 0.919 (1.103)	- 3.090 – 1.251	0.405
Smoking status (Yes vs. No)	- 1.003 (1.129)	- 3.226 – 1.220	0.375
Adequate exercise (Yes vs. No)	- 0.615 (1.091)	- 2.762 – 1.532	0.573
Family history of hypertension (Yes vs. No)	4.796 (1.799)	1.254–8.337	*0.008* ^†^
Per doubling of blood cadmium^a^	1.462 (0.668)	0.147–2.777	*0.029* ^†^
Per doubling of blood lead^a^	- 1.182 (0.803)	- 2.763 – 0.399	0.142
R^2^ = 0.238 (*p* *< 0.001*)			

*p* - value was calculated by multiple linear regression analysis. ^a^logarithm (base 2) transformation of blood cadmium or lead concentration. Those who were taking antihypertensive drugs were excluded. ^†^ *p* < 0.05

**Table 5:** The β coefficient (95% CI) of per doubling of blood cadmium for systolic and diastolic blood pressure according to age stratification after covariates adjustment

Age	Systolic blood pressure	Diastolic blood pressure
< 40	0.623 (−1.252–2.498)	1.429 (− 0.187–3.045)
≥ 40	3.336 (0.572–6.099)*	1.550 (− 0.512–3.612)

The values were adjusted for BMI, fasting blood glucose, dyslipidemia, high risk alcohol drinking habit, smoking status, adequate exercise, family history of hypertension, and per doubling of blood lead. *p* - value was calculated by multiple linear regression analysis. *p < 0.05. Those who were taking antihypertensive drugs were excluded

## Discussion

Studies on the effects of heavy metal exposure on health have been conducted to date. In particular, humans are easily exposed to lead and cadmium that are present not only in the environment, but also in the workplace. Our study aimed to identify the association between heavy metal exposure and blood pressure elevation in workers who were occupationally exposed to lead and cadmium.

We studied 310 workers who were occupationally exposed to lead and cadmium. The GMs of blood lead and cadmium levels of the participants were 5.839 μg/dL and 1.053 μg/L, respectively, which were higher than the mean blood lead and cadmium concentrations of the general male population (2.44 μg/dL and 0.83 μg/L, respectively) based on the report of the Fifth Korea National Health and Nutrition Examination Survey [48]. The results obtained from the measurement of ambient cadmium and lead concentrations in the working environment conducted in 2014 were lower than the time weighted averages of 0.05 and 0.01 mg/m^3^, respectively, which were considered as the exposure criteria by the Korea Ministry of Employment and Labor for the assessment of chemical and physical factors [49]. One of the causes of the higher blood lead and cadmium levels of the participants in this study than those of the general population could be the continuous occupational exposure to lead and cadmium in their workplace. Previous studies also revealed high blood lead or cadmium levels in workers who were occupationally exposed to these heavy metals [50–52].

Blood cadmium levels significantly increased as age and work period groups increased. However, blood lead levels did not significantly changed with age and work period. Our previous studies [25, 53] also showed similar findings that the association of aging with increase in blood cadmium was stronger than that of blood lead. One of the reasons is that the blood lead has shorter half-life and does not fully reflect the total body burden compared to blood cadmium [54, 55]. Also, the blood lead and cadmium levels were higher in current smokers than in current non-smokers (*p* < 0.001 and *p* = 0.017, respectively).

The association between SBP and DBP and heavy metal exposure was examined using simple linear regression analysis. A significant association between blood cadmium level and SBP and DBP was found. However, a statistically significant correlation between blood lead level and blood pressure could not be identified. Moreover, multiple linear regression analysis was used to adjust for other factors, such as age, body mass index, fasting blood glucose, dyslipidemia, high-risk alcohol drinking habit, smoking status, exercise adequacy, and family history of hypertension, those might affect the blood pressure. Similarly, a significant association between blood cadmium level and SBP and DBP was observed after further adjustment for lead exposure. However, a statistically significant relationship between blood lead level and blood pressure was not observed. Our previous studies [25, 53] showed similar findings of stronger association of blood pressure with blood cadmium than blood lead. One of reasons for this result may be small number of subjects. Further study is needed to clarify this difference. In addition, blood cadmium level was significantly associated with SBP, but not DBP, in subjects aged 40 years or older. These findings are compatible with previous studies that showed stronger association of cadmium exposure with SBP than DBP [25, 56, 57].

Blood lead and cadmium levels are valid biomarkers for recent lead [58] and cadmium [59] exposures. Many studies had been performed on the association between cadmium or lead exposure and blood pressure elevation. The hypothesis on the mechanism by which lead exposure could affect blood pressure had been suggested by several studies as follows: changes in the renin–angiotensin system and dysfunction in sodium handling lead to toxic effects in the body upon exposure to low lead concentrations [60]. Lead exposure causes oxidative stress, which induces limitation of nitric oxide availability, elevation of systemic vascular resistance [19], and changes in the activity and production of hormones that regulate the vascular tone [61]. Although studies consistently reported positive associations between bone lead and blood pressure, other researches inconsistently supported the correlation between blood lead and blood pressure [25]. In the present study, the association between blood lead concentration and blood pressure elevation was not identified. Further study is needed to evaluate the association between blood lead concentration and blood pressure.

The exact biological mechanisms by which cadmium exposure leads to blood pressure elevation remains to be precisely unknown. However, these mechanisms include oxidative stress induced by cadmium exposure, resulting in vascular endothelial and smooth muscle cell damage [62], nephrotoxicity by cadmium [63], direct vasoconstriction, autonomic nerve system activation, and vasodilatation inhibition [64]. In the previous study of the authors, the association between blood cadmium levels (GM: 0.788 μg/L) and hypertension in the general Korean population was determined [24, 25], and a significant association between cadmium levels and blood pressure increase was found after adjusting for the blood lead level [25]. In the present study, we also identified a relationship between low-level cadmium exposure (GM: 1.053 μg/L) and elevated blood pressure in workers, similar to the findings in the general population.

This study has some limitations to consider when interpreting the results. First, given that our results were based on cross-sectional analysis, we did not reveal the temporal relationship between heavy metal exposure and blood pressure elevation and/or did not rule out the possibility that an unknown third factor may serve as a confounding variable. Thus, the prospective study is needed further. Second, the identification of the total body burden of exposure to these two heavy metals during the workers’ lifetime is limited because blood lead and cadmium levels are commonly used as indicators to reflect recent exposure; the half-life of cadmium and lead in blood is about 2–3 months [54] and 1 month, respectively [55]. Third, the data were collected from only one hospital and the number of subjects was small. Thus, these facts could have affected results of this study.

However, this study also has some important implications. First, our study presented objective results on the association between lead and cadmium exposure and blood pressure increase in smelting workers. Second, after adjusting for various hypertension risk factors, including blood lead level, this study could still confirm the association between blood cadmium level and blood pressure elevation. Furthermore, this study is based on workers who are occupationally exposed to heavy metals in a smelter. Therefore, long-term follow-up through cohort construction will facilitate the performance of a prospective study on the relationship between heavy metal exposure and blood pressure increase. Additionally, the health effects can be seen more clearly if the measurements used are indicators of cumulative chronic heavy metal exposure, such as bone lead level, rather than a recent one.

## Conclusions

We investigated workers who were occupationally exposed to lead and cadmium to identify the association between exposure to these two heavy metals and blood pressure elevation in a smelter. Although the association between blood cadmium level and blood pressure elevation was confirmed in this study, the relationship between blood lead level and blood pressure increase was not identified. In fact, many workers are exposed to heavy metals, such as cadmium, in the workplace, so studies on health effects caused by heavy metal exposure are continuously needed. Therefore, further research is warranted in the future.

## Abbreviations

CI: Confidence intervals
DBP: Diastolic blood pressure
GM: Geometric mean
SBP: Systolic blood pressure
